# Inflammatory Phenotypes of Bronchiectasis

**DOI:** 10.3390/jpm15100499

**Published:** 2025-10-17

**Authors:** Evangelia Koukaki, Georgia Papaiakovou, Argyri Klironomou, Efthymia Theofani, Andreas M. Matthaiou, Adamantia Liapikou, Nektarios Anagnostopoulos, Grigorios Stratakos, Petros Bakakos, Nikoletta Rovina

**Affiliations:** 1 1st Respiratory Medicine Department of National and Kapodistrian University of Athens, Sotiria Chest Diseases Hospital, 11527 Athens, Greece; gpapaiakovou30@gmail.com (G.P.); klironomouarg@gmail.com (A.K.); aris.anag@yahoo.gr (N.A.); gstratak@med.uoa.gr (G.S.); petros44@hotmail.com (P.B.); nikrovina@med.uoa.gr (N.R.); 2 Cellular Immunology Laboratory, Center for Basic Research, Biomedical Research Foundation of the Academy of Athens, 11527 Athens, Greece; efitheofani@gmail.com; 3 5th Respiratory Medicine Department, Sotiria Chest Diseases Hospital, 11527 Athens, Greece; matthaiou.andreas@gmail.com (A.M.M.); mliapikou@yahoo.com (A.L.)

**Keywords:** bronchiectasis, neutrophilic inflammation, phenotypes, biomarkers, precision medicine

## Abstract

Bronchiectasis is a heterogeneous chronic airway disease traditionally viewed as neutrophil-driven. Emerging evidence demonstrates distinct complex inflammatory phenotypes influencing clinical outcomes, prognosis and therapeutic options. A narrative review was conducted, informed by a structured literature search on PubMed and Google Scholar databases, focusing on inflammatory phenotypes in bronchiectasis. Based on the prevalent cellular population, four distinct phenotypes can be described. The most common is the neutrophilic phenotype, which is associated with frequent *Pseudomonas* infection, severe disease, exacerbations and poor prognosis. Targeted novel-agents for this group such as brensocatib (neutrophil protease inhibition) emerge. The eosinophilic phenotype is defined by elevated blood or sputum eosinophils and is associated with FeNO, IL-5/IL-13 signaling, a possible response to inhaled corticosteroids and biologic agents. The mixed phenotype demonstrates dual neutrophilic and Th2 inflammation. Paucigranulocytic phenotypes remain poorly characterized but with distinct characteristics. Finally, dysregulation of macrophages and lymphocytes as inflammation mediators needs to be studied further. Recent advances have introduced a variety of therapeutic strategies targeting specific inflammatory pathways. Bronchiectasis has a spectrum of inflammatory phenotypes with distinct biological and clinical implications. Recognition and better understanding of inflammatory phenotypes in bronchiectasis may enable opportunities for personalized precision medicine through the transition from empirical management to biomarker-guided, personalized care.

## 1. Introduction

Bronchiectasis is a chronic progressive inflammatory respiratory disease defined by irreversible abnormal dilation of the bronchi, recurrent infections and chronic airway inflammation [1]. The extent of bronchiectasis may range from localization to a single lobe to diffuse bilateral involvement [2]. Once considered a rare disease, it is now increasingly recognized worldwide, with significant variation in prevalence across regions. Patients frequently experience persistent cough, excessive mucus production and recurrent exacerbations, all of which significantly influence patient’s quality of life (QoL) and long-term prognosis, as well as increase healthcare utilization substantially, highlighting its considerable clinical and economic burden [3].

Bronchiectasis is characterized by complex and heterogeneous biological processes, involving a vicious circle of infection, inflammation, mucus retention and structural damage. This interplay leads to diverse clinical manifestations that cannot be adequately explained by a single pathogenic pathway. It is the shared endpoint of numerous underlying conditions, including infectious, autoimmune, allergic, genetic, and inflammatory disorders. However, even after thorough investigation, in a significant number of cases the etiology remains unclear and the condition is classified as idiopathic [4].

Despite its constantly increasing prevalence, which has established it as the third most common lung disease after asthma and COPD (chronic obstructive pulmonary disease), the exact pathophysiological underlying mechanisms remain poorly understood [5]. A complex interplay between inflammation and infectious processes lies at the core of the disease’s pathophysiology, leading to the permanent airway dilation [3]. This inflammatory environment creates ideal circumstances to develop chronic microbial colonization and persistent exacerbations, contributing to disease progression. Distinct inflammatory mechanisms impact clinical manifestations, prognosis and treatment, with the neutrophilic phenotype being the most common [5]. However, growing evidence supports the presence of distinct inflammatory phenotypes, such as neutrophilic, eosinophilic, mixed type and paucigranulocytic profiles, each with differing clinical outcomes and therapeutic options and responses. Understanding these phenotypes enables precision medicine, as it allows for the use of targeted therapies to improve patient outcomes.

Disease heterogeneity is the most challenging aspect of bronchiectasis and is directly linked to unmet personalized treatment needs. Despite advances in knowledge and therapeutic options, current treatment is often guided by symptoms or exacerbation history rather than underlying biology. This gap underscores a major challenge: the lack of validated biomarkers and stratification tools to link distinct subsets of bronchiectasis patients to personalized care. Improved understanding of distinct inflammatory phenotypes may offer new insights into disease mechanisms and potential therapeutic avenues. This review explores these inflammatory phenotypes, their clinical relevance, and their implications for personalized medicine.

## 2. Materials and Methods

This narrative review was informed by a comprehensive literature search of electronic databases, including PubMed and Google Scholar. Due to the nature of the topic and the heterogenicity of the related literature, a narrative review rather than systematic was considered more appropriate for this topic. The aim was to synthesize current knowledge and emerging concepts related to inflammatory phenotypes in bronchiectasis rather than to perform a quantitative analysis.

The starting point of this research is placed in January of 2025. The search strategy incorporated a combination of key terms, including but not limited to “bronchiectasis” “inflammation”, “neutrophils”, “exacerbations” and “inflammatory phenotypes” ensuring the comprehensive identification of relevant studies. Titles and abstracts were screened for relevance, followed by a full-text review of selected studies.

Studies were included if they met the following criteria: (1) published in peer-reviewed journals, (2) written in English, (3) focused on bronchiectasis and inflammation and (4) published the last 10 years. Conference abstracts, unpublished reports, or irrelevant topics were excluded. The selection process involved evaluating both original research articles and relevant review papers, which were systematically shortlisted based on their relevance to the topic. This review is based solely on previously conducted research and does not include any new experiments or studies conducted by the authors.

## 3. Etiology and Pathophysiology of Bronchiectasis

Bronchiectasis represents the end-point condition that emerges from a variety of inherited and acquired disorders that remodel and dilate the airways. Inherited and acquired disorders can lead to bronchiectasis. Regarding inherited disorders, cystic fibrosis (CF) is the most common genetic disease linked with bronchiectasis followed by primary ciliary dyskinesia, a1 antitrypsin deficiency, primary immunodeficiencies and other rare genetic defects like Kartagener syndrome [4,6,7,8]. Regarding acquired disorders, respiratory infections (tuberculosis or non-tuberculosis), immunodeficiencies, autoimmune/collagen tissue disease, chronic obstructive pulmonary disease, airway obstruction, post-radiation and traction in fibrotic parenchymal disease are only some of the possible etiologies. Despite great interest in the diagnostic work-up, a substantial proportion of cases are still Labeled idiopathic [9].

A recent analysis by Gomez et al. highlights the significant geographic heterogeneity in the prevalence of bronchiectasis etiologies across different countries [10]. Idiopathic bronchiectasis is the most common cause in Northern Europe, whereas in India and Eastern Europe, post-infectious and mainly post-tuberculosis bronchiectasis are more common [10]. Pulmonary infections are a key factor of acquired bronchiectasis. Frequent or severe lower-respiratory system childhood infections as well as bacterial bronchitis (severe bacterial infection with chronic wet cough with duration more than 4 months) predispose to adult bronchiectasis [3]. 

The bacterial load, rather than the specific pathogen, seems to determine the intensity, chronicity and progression of the inflammatory response [3]. The most commonly identified microorganisms are *Haemophilus influenzae*, *Pseudomonas aeruginosa*, *Streptococcus pneumoniae* and *Staphylococcus aureus* [11,12]. *P. aeruginosa* is associated with the worst disease progression [3]. Additionally, *Aspergillus fumigatus* and the associated Allergic Bronchopulmonary aspergillosis (ABPA) are recognized causes, especially in patients already suffering from asthma, or CF [13].

Notably, up to half patients with COPD and one fifth with asthma have bronchiectasis without any other identifiable cause [10,14].

Pathophysiologically, this diverse aetiologic spectrum converges on a shared cascade of mucociliary impairment, endothelial dysfunction, inflammation, chronic infection and structural injury [2]. Mucociliary dysfunction, impaired mucus clearance and retention of airway secretions increase the susceptibility to recurrent infections. Infection, then, triggers inflammatory responses and release of an exuberant neutrophilic and cytokine-mediated response, eventually causing an airway remodeling and the characteristic dilated pattern of bronchiectasis [15].

**Figure 1:**
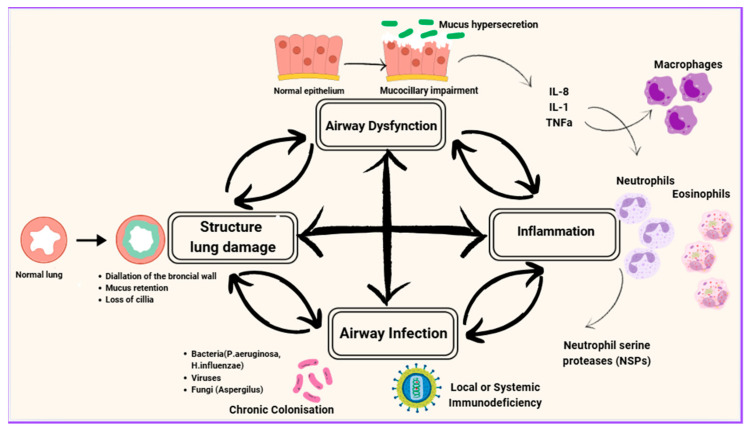
Pathophysiology of bronchiectasis.

Cole initially described this as a “vicious circle,” where each factor perpetuates the others in a continuous cycle [16], while Flume et al. revised the traditional cyclical model, proposing the “vicious vortex” as a more accurate representation of these dynamic interactions among mucociliary dysfunction, infection, inflammation and tissue destruction [2] (Figure 1). The interactions between these components are very complex and do not always follow a linear progression. Each factor seems to influence all the others, creating a pathway that cannot easily be dealt with by targeting a single factor. Instead, complex sequences occur among all the factors, forming discrete yet interconnected pathophysiological pathways [17].

Different molecular biomarkers in the blood and sputum of each patient, as well as their quantity, appear to be particularly useful in assessing the severity and more importantly in the classification of bronchiectasis. For example, neutrophil elastase (NE) is the main biomarker associated with the pathophysiology of bronchiectasis and is found in the neutrophil phenotype. Other substances such as mycins, antimicrobial peptides and matrix metalloproteinases, as well as bacterial load, also characterize this phenotype, and their presence indicates increased severity of the condition [18].

The pathophysiology of bronchiectasis involves highly complex processes. A deeper understanding promises more precise, targeted interventions capable of disrupting the vicious vortex cycle and altering the course of bronchiectasis.

The figure depicts the vicious vortex of bronchiectasis, highlighting the cyclical interplay between infection, inflammation, airway dysfunction (mucus hypersecretion and mucociliary impairment) and structural lung damage. Airway infection with bacteria (e.g., *Pseudomonas aeruginosa*, *Haemophilus influenzae*), viruses or fungi (e.g., *Aspergillus*) triggers recruitment of inflammatory cells including neutrophils, eosinophils and macrophages. Neutrophil proteases such as neutrophil elastase, eosinophil-derived cytotoxic proteins, and macrophage-mediated immune responses contribute to epithelial injury and alteration of mucus properties. Impaired mucociliary clearance and mucus hypersecretion promote microbial persistence, while cumulative epithelial injury and tissue remodeling lead to irreversible structural lung damage. These processes perpetuate one another, forming a self-reinforcing “vicious vortex” that drives disease progression and clinical worsening.

## 4. Inflammatory Phenotypes of Bronchiectasis

Phenotyping in bronchiectasis traditionally relies on observable clinical and biological features such as inflammatory cell predominance, microbiological profiles or comorbidity associations. In contrast endotyping reflects distinct subgroups with shared underlying molecular mechanisms that drive these traits, such as specific cytokine pathways. Recognizing phenotypes and endotypes is central to translational relevance: while phenotypes group patients by presentation, endotypes provide insight into disease mechanisms. This distinction allows molecular stratification, which enables more precise clinical decision-making by linking disease mechanisms to targeted therapies, thereby advancing precision medicine in bronchiectasis.

Distinct phenotypes based on the predominantly implicated cells in bronchiectasis are described below.

### 4.1. Neutrophilic Phenotype

#### 4.1.1. Neutrophilic Dysfunction and NETs

Neutrophilic inflammation is the most common and well-characterized inflammatory pattern in bronchiectasis. It is the central mediator in both vortex and vicious circle models of disease progression [2,16]. This phenotype is characterized by a chronic and excessive inflammatory response driven by neutrophil recruitment and activation. Neutrophils are the dominant inflammatory cells both in stable state and during exacerbations. Neutrophilic accumulation results from the host’s response to microbial attack, followed by the release of pro-inflammatory mediators such as interleukin (IL)-1β, IL-8, IL-17, leukotriene B4 and tumor necrosis factor-α (TNF-α), which are responsible for the accumulation and degranulation of the immune cells [19,20]. The released proteins enhance and promote further inflammation, resulting in disruption of the epithelial repair mechanisms and recurrent lung injury. Neutrophilic phenotype is strongly linked to chronic infections and worsening disease progression [21,22]. Sputum neutrophilic inflammation is associated with disease severity, duration and FEV1% (forced expiratory volume in 1 secons) decline [23].

Furthermore, Angrill et al., in a case–control study, showed that BAL (bronchoalveolar lavage) samples from bronchiectasis patients exhibited significantly elevated levels of NE, myeloperoxidase, TNF-α, CXCL-8 and IL-6, compared to controls [24]. In this study this inflammatory profile was correlated with higher bacterial loads and an increase in neutrophil accumulation; thus, these proteinases are responsible for recruiting and activating inflammatory cells at the site of the infection.

The disease pathology is not only driven by the excessive gathering of the neutrophils, but also by their dysfunction. The neutrophilic apoptotic process is delayed, impairing bacterial clearance, a pattern that also is present in exacerbations [25]. Notably, in this phenotype, systemic IL-8 levels have been found to be elevated [26]. High levels of IL-8 and TNF-a appear to inhibit the apoptotic process [27]. While neutrophils are essential for host defense, their prolonged activation leads to excessive release of proteases, such as NE and myeloperoxidase, proteinase 3 (PR3), cathepsin G (CatG) and Nonstructural protein 4 (NSP4) [22]. These proteases contribute to tissue destruction, impair mucociliary clearance and drive airway remodeling. During this process, reactive oxygen species (ROS) are simultaneously generated, enhancing the lung injury [22].

Neutrophilic extracellular traps (NETs)—a network of extracellular fibers from DNA, histones and bactericidal proteins designed to neutralize pathogens—are also formed by activated neutrophils. Excessive NET formation, however, promotes tissue damage and persistent airway inflammation. It has been strongly associated with disease severity and antibiotic response in this type of patient [23,28]. NE is a dominant mediator in this inflammatory process. This pro-inflammatory serine protease slows ciliary-beat frequency and enhances mucus secretion [29]. It accumulates to high concentrations in sputum of patients with neutrophilic lung diseases, including but not limited to bronchiectasis, even in the absence of bacterial Colonization [30,31]. Normally, endogenous inhibitors, such as secretory leukoproteinase inhibitor ant α1 antitrypsin delivered by bronchial epithelium and by serum accordingly, inhibit the action of NE [32]. Furthermore, DNA formation from NETs can directly inhibit the NE function and indirectly alter how NE inhibitors function [32,33]. Molecules produced by bronchial epithelium like syndecan-1 can bind in NE and deactivate it [32,33]. Interestingly, in bronchiectasis, these defending mechanisms seem to be insufficient, because the release of NE is abundant, as indicated by high levels of this protein detected in sputum and BAL [32]. Chalmers et al. linked elevated NE levels in BAL with increased bacterial load, more extensive radiological damage, and *P. aeruginosa* colonization, highlighting NE as a potential biomarker for disease severity [21]. Point-of-care NE assays (eg NEATstik) can measure sputum NE levels and identify patients at a higher risk of exacerbation [34].

#### 4.1.2. Infections and Exacerbations

Chronic infection is the most frequent trigger of the vicious vortex model and is strongly associated with the neutrophilic phenotype. Among pathogens, chronic *P. aeruginosa* colonization is especially linked to this phenotype, which is the most identified phenotype in all cohorts [2,22]. Sputum neutrophilia is increased markedly with high bacterial loads and specific bacterial types. Patients with *P. aeruginosa* have greater sputum neutrophilia and more severe airflow limitation (lower FEV1) compared with patients infected with other pathogens [35].

*P. aeruginosa* infection/colonization is associated with more severe disease and worse outcomes such as increased risk for hospitalization, worsening QoL and frequent exacerbations [17,31,36,37]. It is isolated in the sputum of both CF and non-CF patients [38] and it is associated with both steady disease and exacerbations, and it is an important marker that predicts the risk of future exacerbations, worse quality of life and increased patient mortality [11,38,39,40,41]. In a study by Tunney et al., lung microbiota of patients with bronchiectasis were analyzed both in stable conditions and in exacerbations. *P. aeruginosa* was demonstrated to be among the dominant pathogens during stable condition (10 out of 40 individuals with aerobic bacteria) and the most dominant pathogen in exacerbations (5 out of 14 at the start of the exacerbation), although only in 1 individual were the *Pseudomonas* detected at the end of the exacerbation, after the use of antibiotics [11]. It is worth mentioning that *P. aeruginosa* was detected in 3 out of 13 patients of these patients during clinical stability, suggesting that in some individuals, it may persist chronically even outside of exacerbation episodes [11]. It has been proposed that changes in microbiota strains or in bacterial load could be the trigger of exacerbations. However, in this study, no significant changes in sputum bacterial density or community diversity were observed before and after treatment of exacerbations, suggesting that alterations in lung microbiota composition or increase in bacterial load are not solely responsible for exacerbations in bronchiectasis patients. Exacerbations in bronchiectasis are likely driven by a combination of host immune responses, infections (viral or bacterial) and dysregulated airway inflammation. These findings highlight the multifactorial nature of exacerbations.

Infections from *P. aeruginosa* as well as from *H. influenza* can directly create tissue damage and survive in the microenvironment of the lung, inhibiting factors that contribute to mucociliary impairment [42]. Furthermore, a key survival strategy is the formation of biofilms. More specifically, both *H. influenza* and *P. aeruginosa* create biofilms and manage to utilize extracellular DNA from neutrophils, destroying the NET formation and thus surviving phagocytosis [42]. In this way, protection is provided both from host defenses and also from antibiotic treatments [42,43].

Exacerbations demonstrate a key role in disease progression and mortality [11]. Exacerbations—defined as worsened symptoms for over 48 h and requiring a change in treatment—have a negative effect on lung function decline and quality of life, as well as on mortality. As in COPD, a patient’s history of prior exacerbations is a strong predictor for future exacerbations [44]. Patients who had an exacerbation have nearly twice the risk for another compared to those who remain stable [45]. The majority of therapeutic interventions are aimed at reducing exacerbation rates. Remarkably, half of the European bronchiectasis patients experience at least two exacerbations per year, while one third require hospitalization. Although the exact triggers are not completely clear, chronic infections are regarded as one of the main triggers of exacerbations, with bacteria playing a dominant role [31]. Neutrophilic phenotype is strongly associated with higher risk of exacerbations, but many other factors are involved in the inflammatory process [31].

Several studies have explored the relationship between chronic infections and frequency of exacerbations. In a study by Gao et al., 120 bronchiectasis patients with at least one exacerbation in the last 12 months were examined, and at least one bacterium was identified in 86% of the patients [46]. NE, proteinase-3, IL-1β and CXCL8 were found increased during exacerbation, indicating their association with bacterial and bacterial-plus-viral exacerbations [46].

Chronic *P. aeruginosa* infection is associated with worse outcomes. However, a large observational study demonstrated that mortality was increased only in *P. aeruginosa* patients who experience at least 2 exacerbations per year [39]. The study also included individuals infected with P. aeruginosa who did not experience frequent exacerbations, suggesting heterogeneity in clinical impact. Further research is needed to clarify the role of *P. aeruginosa* in disease worsening and mortality outcomes in these patients.

Viral infections—including rhinovirus, influenza and respiratory syncytial virus—are increasingly recognized as important contributors to exacerbations, and viruses are present in up to 50% of them. Exacerbations therefore reflect a multifactorial process involving both bacterial and viral pathogens, together with dysregulated host inflammatory responses and air pollution [47].

#### 4.1.3. Systemic Neutrophils

Although the predominant neutrophilic activity is in the airways, a few studies have demonstrated the presence of systemic inflammation as depicted by circulating neutrophils and the increase in systemic inflammatory markers both in stable disease and in exacerbations [28]. The higher the airway levels of bacterial loads are, the more intense the systemic inflammation becomes, and simultaneously the risk of exacerbation is elevated [21]. That systemic inflammation has been associated with disease severity and is linked with an increased risk of cardiovascular and metabolic diseases [48]. In addition, Saleh et al. conducted a study that demonstrated the heterogeneous profile of systemic inflammatory proteins and the relationship between them and disease severity, pointing to the presence of acute-phase serum proteins and specifically fibrinogen in severe cases of bronchiectasis [49]. Another study pointed out the high concentration of TNF-a in the plasma of patients with non-CF bronchiectasis, indicating the high accumulation of serum neutrophils.

Serum neutrophils in bronchiectasis seem to be dysfunctional; they are characterized by extended survival and impaired phagocytic action [25]. In fact, it was observed that in healthy controls, phagocytosis and bacterial elimination was much greater than in patients with mild to severe bronchiectasis. The reason for this phenomenon is not yet clear; the inflammatory milieu of bronchiectasis and intrinsic reprogramming of neutrophils might influence this process. Additionally, the use of antibiotics during acute infectious exacerbations enhances the phagocytic function of neutrophils, leading to its partial restoration to levels observed in the stable phase of the disease [25]. However, neutrophil function remains impaired compared to individuals who have recovered from community-acquired pneumonia with the same antibiotic treatment. Moreover, blood neutrophil counts were significantly lower in these individuals, while their functional capacity was superior [25]. Systemic inflammation and its role in bronchiectasis’ pathophysiology is a field that needs to be further studied in order to become the target of new personalized therapeutic approaches.

Lastly, another factor of particular interest is Neutrophil to Lymphocyte Ratio (NLR). This ratio measured in peripheral blood is a biomarker that presents the relationship between the innate (neutrophils) and adaptive cellular immune response (lymphocytes) during illness and pathological states; therefore, it is a widely used inflammatory biomarker [50]. It has been observed that NLR values are greater in patients with exacerbations than in healthy controls, and specifically in those with positive sputum cultures. Thus, NLR has been proposed as an important biomarker predicting bacterial colonization in patients with exacerbations [51].

### 4.2. Eosinophilic Phenotype

While neutrophilic inflammation is the predominant and most studied mechanism in bronchiectasis, a subset of patients—estimated to be approximately 20%—exhibit eosinophilic inflammatory profile. This can be secondary to severe asthma or other eosinophilic diseases, or bronchiectasis might demonstrate an eosinophilic pattern of inflammation on their own [18].

#### 4.2.1. Definition, Pathophysiology and Biomarkers

Eosinophilic inflammatory type is defined as ≥3% sputum cells [35,52] or as ≥300 cells/μL in blood [18,53] in the absence of comorbidities that independently raise blood eosinophil counts (BEC). Reported etiologies include a frequency order preceding infection, idiopathic, immune deficiency, systemic disorder, inflammatory bowel disease and genetic disorder [54].

Eosinophils are important mediators in lung diseases such as asthma and COPD. They regulate immune responses in a pro-inflammatory way by releasing cytotoxic granule proteins, cytokines, chemokines and lipid mediators and in an immunomodulatory way by promoting Type 2 inflammation [55,56,57]. In bronchiectasis, increased sputum eosinophils are related to greater bronchodilator reversibility and higher levels of FeNO, IL-13 [52] and an increase in sputum Eosinophil Peroxidase (EPX) [58]. EPX has been proposed as a specific biomarker for eosinophilic activity (especially found in severe disease and frequent exacerbations), while FeNO and blood eosinophil count provide accessible markers for clinical stratification [58].

#### 4.2.2. Microbiome and Exacerbations

Evidence on the clinical impact of eosinophilia is conflicting. Shoemark et al. associated high BEC (≥300 blood eosinophils/μL) with *Streptococcus*- and *Pseudomonas*-dominated microbial clusters and increased exacerbation risk, while low BEC (≤100 cells/μL) was related to *Haemophilus*-dominated profile. The study’s findings also demonstrate that eosinophilic type serves as a risk factor for exacerbation in patients with *Pseudomonas infection* [53]. Its predominance is linked to severe disease characterized by worse lung function, increased antibiotic use, higher frequency of exacerbation and all-cause mortality [59]. On the other hand, *Haemophilus*- and *Streptococcus*-dominant clusters are not associated with disease severity [59,60]. The multicohort study of Shoemark et al. reported lower mortality rates but shorter time to exacerbation in patients with eosinophilia (either 100–299 cells/μL or ≥300 cells/μL) [53]. In contrast to these findings regarding exacerbations, Wang et al. showed that patients with BEC > 100 cells/μL had milder disease with lower rates of exacerbation, while patients with BEC < 100 cells/μL had significantly more severe disease [61]. These findings were consistent with those of the multicenter study of Garcia et al. that demonstrated a significant U-shaped relationship between BEC and bronchiectasis severity [54]. The U-shape relationship described peaks of exacerbation rates both at the eosinopenic (<50 cells/μL) and the eosinophilic (>300 cells/μL) subgroups, with the lowest prevalence of exacerbations reported in middle-range BEC groups. Interestingly, the eosinopenic group was characterized by more severe disease compared to the eosinophilic group. This phenomenon may be associated with the higher levels of *Pseudomonas* colonization that was observed in the eosinopenic group [54].

These discrepancies suggest that eosinophilia may identify heterogeneous subgroups, with outcomes influenced by coexisting microbiological profiles and comorbidities. Microbial diversity plays a key role in bronchiectasis pathophysiology. Data suggests that diminished microbial variety correlates with disease severity, lower quality of life and higher mortality [60].

#### 4.2.3. Eosinophilic Airway Comorbidities and Overlap Syndromes

Eosinophilic bronchiectasis often coexists with asthma, COPD and ABPA. Chronic Obstructive Pulmonary Disease (COPD) serves as an eosinophilic bronchiectasis etiology in 5.6% of patients having 100–299 eosinophils/μL and in 9.8% of patients having ≥300 eosinophils/μL [53]. The coexistence of bronchiectasis and COPD is an entity described as Bronchiectasis–COPD (BCOS) overlap syndrome. It is suggested that BCOS is associated with elevated BEC levels and the presence of eosinophilic markers and Th2 cytokines, implying that BCOS inflammation is driven by eosinophils [62,63]. Regarding exacerbation and annual hospitalization rates in patients having the syndrome, findings show an increase as BEC levels rise [64].

Asthma is one of the most common comorbidities of bronchiectasis [65]. Research indicates that coexistence of bronchiectasis and asthma is associated with eosinophilia [66]. The dual pathology of asthma and bronchiectasis has been linked to higher blood eosinophil count compared to bronchiectasis alone [67]. However elevated BEC does not appear to significantly correlate with the exacerbation rate in patients having both the diseases [66]. Interestingly, Zhang et al. demonstrated that allergic asthma increases the risk of developing bronchiectasis, but no causal relationship was found in the opposite direction [68]. Alongside this, the study revealed genetic links between the two diseases.

*Aspergillus* can manifest in bronchiectasis patients in many forms: Allergic Bronchopulmonary aspergillosis (ABPA), *Aspergillus* Sensitization (AS) and increased IgG levels against *Aspergillus*, indicating exposure or infection. ABPA and AS are linked to Th2-driven inflammation with IL-5-mediated eosinophil recruitment and IgE production by B cells [69]. A retrospective cohort study has demonstrated that patients with ABPA and extensive bronchiectasis exhibited poorer lung function, heightened immunological activity and an increased risk of ABPA exacerbation [70].

### 4.3. Mixed Neutrophilic–Eosinophilic Phenotype Bronchiectasis

Although bronchiectasis has long been viewed as a predominantly neutrophilic disease, emerging evidence indicates that a substantial proportion of patients displaying a mixed—neutrophilic and type 2 (T2) inflammation—cell type is increasingly recognized.

Mixed inflammation is defined by the co-expression of neutrophilic (e.g., NE, IL-8, TNF-a) and eosinophilic markers (IL-5, IL-13, FeNO). In the European Multicenter Cohort Study by Choi et al., which enrolled 199 patients. Nearly one-third (31.2%) of patients were identified as having mixed neutrophilic–eosinophilic type, accounting for the second most frequent cluster, after the mild neutrophilic cluster [71]. Patients were classified in four clusters according to their inflammatory endotype as follows: cluster 1: milder neutrophilic inflammation; cluster 2: mixed—neutrophilic and T2; cluster 3: most severe neutrophilic; and cluster 4: mixed—epithelial and type 2 [71]. In terms of the microbiome, clusters 2 and 3 were dominated by *Pseudomonas*, which was linked to reduced microbial diversity and increased exacerbation rates [71]. No differences between blood eosinophil counts were observed among the four clusters.

Similarly, Tsikrika et al., in a study that enrolled forty patients, identified the mixed neutrophilic–eosinophilic phenotype in 12.5% of participants [52]. Mixed phenotype was the least common, while neutrophilic and paucigranulocytic were the most prevalent. Substantial neutrophilic involvement (in the neutrophilic and mixed type) was linked to greater bronchial destruction in HRCT, higher levels of sputum IL-8 and lower bronchodilator reversibility. Additionally, the presence of high levels of eosinophils (in eosinophilic and mixed type) was associated with increased levels of sputum IL-13 and FeNO levels and greater bronchodilator reversibility [52]. Notably, *Pseudomonas* chronic colonization was related to neutrophilic inflammation and increased levels of IL-8 in sputum [52]. 

Mixed neutrophilic–eosinophilic bronchiectasis seems to represent a distinct biological endotype rather than a transitional state. This phenotype is characterized by dual inflammatory drivers, higher exacerbation risk and potential responsiveness to combined therapeutic strategies. Further studies are needed to guide management in this subgroup, as specific biomarkers may help stratify patients for personalized multimodal therapy.

### 4.4. Paucigranulocytic Bronchiectasis

A smaller proportion of patients with bronchiectasis exhibit minimal granulocytic inflammation. Paucigranulocytic phenotype is characterized from the presence of <60% neutrophils and <3% eosinophils in sputum with no clear dominance of other inflammatory cell types. In bronchiectasis, there have been attempts to extrapolate data from the paucigranulocytic phenotype of asthma (PGA) as there is limited data available for this phenotype. PGA is a relatively common phenotype, affecting 31–47% of the total asthma population [72,73]. Regarding the inflammatory mediators, PGA patients express notably reduced levels of mediators compared to patients with neutrophilic or eosinophilic asthma. Lastly, in terms of treatment, a significant proportion of patients remain with uncontrolled asthma.

In the study by Tsikrika et al., 15 out of 44 patients (37.5%) exhibited paucigranulocytic phenotype based on sputum cell counts [52]. Expression of IL-8 and IL-13 was significantly lower in this phenotype compared to the others, as well as the levels of FeNO, a biomarker usually associated with high eosinophil count in asthma, and observed in patients with steady bronchiectasis in comparison with healthy individuals [74]. Paucigranulocytic phenotype seems to be associated with milder disease in HRCT imaging and milder clinical manifestations. However, currently there are not enough studies proposing this endotype as a distinct entity or just a low-inflammatory burden state. Although a mixed epithelial cell phenotype has been proposed, as well as a mild neutrophilic phenotype and other cell types seemingly involved in the diseases pathogenesis, there is a lack of classification of these patients [71,75]. Consequently, this knowledge gap could be the subject of new studies both in terms of the clinical manifestations of the disease and the potential treatment approaches for these patients. Paucigranulocytic phenotype highlights the heterogeneity of bronchiectasis.

### 4.5. Macrophage- and Lymphocyte-Related Inflammation in Bronchiectasis

The role of macrophages in bronchiectasis is less explained than the role of neutrophils and eosinophils.

Macrophages play a crucial role in the immune system beyond responding to pathogens. They also regulate airway neutrophil levels by releasing inflammatory mediators such as TNF-α and endothelin-1 (ET-1) and by facilitating apoptotic cell clearance through the efferocytotic process [5,75,76]. More specifically, apoptotic cells have specific surface targets, mostly phosphatidylserine (PS), in order to interact with the macrophages, and start the phagocytic process. In bronchiectasis, an increase in apoptotic cells, along with impaired phagocytosis, indicates dysfunctional macrophage activity [5,25,75]. Additionally, it has been proposed that NE can degrade the PS receptor, further disrupting apoptosis [75]. This decreased clearance can lead to further release of inflammatory mediators and ROS, promoting airway inflammation and enhancing tissue damage [5]. In bronchial biopsies, specifically in lamina propria of patients with bronchiectasis, an abundance of macrophages compared with normal controls has been identified [77].

Very interesting insights are demonstrated in a study by Zheng et al. [76]. In a study by Zheng et al., macrophages appear to be increased in bronchiectasis patients (almost double than controls), specifically in those with sputum production [76]. Significant association between TNF-α-positive cells and macrophages in bronchiectatic airways was demonstrated, suggesting that airway macrophages may be a primary source of TNF-α in the airways. Thus, the neutrophil accumulation, and the following clinical manifestations of the severe disease, are strongly regulated through the action of macrophages. Taking these results into consideration, it could be proposed to consider the macrophage count in airways as a sensitive biomarker in diseases severity [76].

Lymphocytes demonstrate a significant role in bronchiectasis’ pathogenesis, particularly T-helper (Th, Th-1, Th-2) cells and T-regulatory (T-regs) cells. It is worth noting that B and T lymphocytes have been detected in biopsies from bronchial samples from adults and children with bronchiectasis, indicating the importance of immune cells in disease pathogenesis [78]. A histological increase in CD4+ T cells and CD68+ macrophages in the bronchial mucosa from post-infective bronchiectasis was observed in a study by Gaga et al. [79]. Elevated levels of CD4+ cells have been associated with systemic conditions like rheumatoid arthritis, Sjogren syndrome and ulcerative colitis, all of which have been linked to bronchiectasis [80]. Interestingly, according to a study from Silva et al., there is a predominance of T-helper cells over T cytotoxic ones in these inflammatory conditions [81]. Lymphocytic inflammation (CD8+ cells) was detected in the majority of patients with bronchiectasis caused by diverse etiologies, as it was shown in a study by Eller et al. [82].

Different T cells play distinct roles. In neutrophilic inflammation, TH17 cells are important, as they promote neutrophil activation through the production of interleukins 17A and 23. In contrast, TH2 cells drive eosinophilic inflammation, and elevated levels of type 2 (T2) inflammation are also observed in chronic *Pseudomonas* infections. Finally, a deficiency of TH1 cells—which normally protect against bacterial infections—contributes to the disease’s pathophysiology [83].

In summary, macrophage and lymphocyte involvement represents an additional layer of immunopathology in bronchiectasis. Their mechanistic contribution highlights the need for deeper immunophenotyping and exploration of novel therapeutic targets beyond granulocytes.

## 5. Current Treatment Approaches

Managing bronchiectasis is a very complicated subject, due to the complex pathophysiology and the heterogenous etiology of this disease. Treatment approaches by phenotype are summarized in Table 1.

### 5.1. Antibiotic Treatment (Oral- and Airway-Targeted)

Guidelines suggest a 14-day antibiotic treatment for acute exacerbations, followed by eradication antibiotic treatment for patients with bronchiectasis and new bacterial isolation limited to *P. aeruginosa* [1]. Long-term antibiotics are advised for patients with ≥3 exacerbations per year, typically starting with inhaled antibiotics for chronic *P. aeruginosa* infections. Macrolides are administrated, if the inhaled antibiotic is contraindicated or difficult to tolerate and additionally to the inhaled antibiotic, in patients with *P. aeruginosa* infection and frequent exacerbations despite the primary therapy or in frequent exacerbators with non *P. aeruginosa* pathogens [1].

In several studies, patients with bronchiectasis and at least one exacerbation in the previous year underwent treatment with erythromycin or azithromycin for 12 months or a higher dose of azithromycin for 6 months. The studies demonstrated that both erythromycin and azithromycin had spectacular results in terms of reducing relapses, sputum production and generally improving the patients’ QOL; however, a significant increase in macrolide resistant bacteria was observed in 88% of the treatment group of patients compared to 26% on placebo (BAT study) [84,85]. To prevent macrolide-resistant NTM, NTM infection must be ruled out before starting treatment [84,86].

Inhaled antibiotics, as observed in the results of the large RESPIRE and ORBIT trials, show a small but significant decrease in exacerbations, are well-tolerated and reduce bacterial load; however, they show no improvements in symptoms or quality of life [87,88,89]. There is still no evidence for the effectiveness of macrolides administrated for longer than 12 months [23].

An interesting study by Fouka et al. reported that prophylactic treatment with low-dose clarithromycin reduced both systemic and local Th17 response, which is a very crucial factor of neutrophilic inflammation in non-CF patients [77]. Studies also indicate that antibiotic treatment lowers the NE, IL-8 and TNF-a levels, such as the neutrophil count, but does not significantly affect neutrophil apoptosis, as was expected, highlighting the complexity of bronchiectasis pathology [27].

### 5.2. Inhaled Corticosteroids

Current guidelines on bronchiectasis management do not recommend inhaled corticosteroid (ICS) use as a treatment, except in cases of asthma, ABPA and/or COPD comorbidity [1]. However, it has been demonstrated that ICS use decreases the number and the severity of exacerbations in patients with eosinophilic bronchiectasis [54]. A similar tendency has been reported regarding hospitalization frequency in patients with elevated BEC and treated with ICS, compared to patients with low BEC who were not using ICS. Notably, neither ICS users with elevated BEC nor ICS non-users with elevated BEC exhibit a significantly increased mortality risk compared to patients with bronchiectasis, lacking eosinophilic inflammation and not treated with ICS [90]. Furthermore it has been observed that levels of CD3+ and CD4+ T cells are reduced in bronchiectasis patients under ICS patients [79].

Although inhaled corticosteroids (ICS) are a cornerstone for the treatment of numerous pulmonary disorders, in most cases of bronchiectasis, especially in neutrophilic phenotypes, they do not have this impact. The lack of evidence supporting the effectiveness of inhaled corticosteroids (ICS) in bronchiectasis suggests that their use should be reconsidered, particularly in the absence of upper-airway hypersensitivity. Moreover, the combination of ICS with long-acting β2-agonists (LABA) has not shown significant benefits over formoterol alone. In fact, patients treated with budesonide and formoterol experienced worsening symptoms, such as increased dyspnea and cough, with no improvement in bacterial load in sputum [86].

Furthermore, ICS use has been associated with adverse effects, including increased risk for infections, in particular with *P. aeruginosa* and non-tuberculous mycobacteria, as well as an increased risk of pneumonia. The impact of ICS on *P. aeruginosa* infection remains unclear. While older studies found no correlation between ICS use and *P. aeruginosa* colonization, a more recent study involving 264 patients from two Danish hospitals reported a higher prevalence of *Pseudomonas* infections among ICS users (24 out of 33 infected patients were on ICS, compared to 9 who were not) [91,92].

Given the uncertain benefits of ICS in bronchiectasis, their potential adverse effects, and the predominance of the neutrophilic phenotype in this condition, ICS therapy does not appear to be a viable option for the pharmacological management of all bronchiectasis patients, but it may be of benefit for the eosinophilic phenotype.

### 5.3. Biologic Agents for Targeted Treatment

Biologic agents targeting the eosinophilic interleukin 5 (IL-5) or the α chain of the receptor of IL-5 (IL-Rα), mepolizumab and benralizumab, respectively, are novel treatment options for eosinophilic bronchiectasis. Their use in patients with refractory disease have shown significant results including improved lung function, reduced symptoms, exacerbation frequency and eosinophil count [93].

Brensocatib is an oral reversible inhibitor of dipeptidyl peptidase 1 (DPP1) which prevents activation of neutrophil serine proteases. NE has been strongly linked with increased disease severity and risk of exacerbations. In a phase 3 RCT ASPEN trial, adults and adolescents with bronchiectasis were randomized to receive brensocatib 10 or 25 mg vs. placebo. Once-daily treatment led to a lower annualized rate of pulmonary exacerbations and less FEV1 decline [94,95]. Skin hyperkeratosis and periodontitis have been described as complications.

The findings of phase 3 ASPEN trial build upon earlier phase 2 data from WILLOW trial. The WILLOW study demonstrated a 40% lower risk of exacerbation, a prolonged time to first exacerbation and less decline in FEV1 in the brensocatib groups compared to placebo [96]. Furthermore, post hoc analyses revealed effectiveness in lowering yearly exacerbation rates regardless of the coexistence of eosinophilic inflammation [97,98], *P. aeruginosa* colonization, disease severity or concomitant macrolide therapy [98]. Subgroup analyses further highlighted a low number needed to treat and a negative number needed to harm, suggesting a beneficial safety and efficacy profile [99]. Identifying patients with bronchiectasis and increased neutrophilic inflammation based on NET or NE levels could enable targeted therapies [34]. Interestingly, a post hoc analysis revealed that brensocatib reduces sputum activity of neutrophil proteases, including NE [97].

### 5.4. Mucoactive Therapy

Beyond pharmacological interventions, physiotherapy and airway clearance techniques remain one of the mainstays of bronchiectasis management and are essential for improving mucus clearance. Mucus plays a central role in the pathophysiology of the disease: its abnormal accumulation provides a niche for persistent infection and inflammation, thereby fueling the vicious vortex [100]. Inflammatory cells further alter mucus properties; neutrophils release DNA and proteases that increase mucus viscosity and impair clearance, while eosinophils contribute through secretion of cytotoxic granules and protein [100].

Physiotherapy along with hypertonic saline 7%, inhaled mannitol, DNase, acetylcysteine and carbocisteine plays an important role in mucus clearance. Although it has been proposed as a long term treatment (>3 months) in patients with poor QoL that standard airway clearance techniques have failed, their role during exacerbations is controversial [1]. Some studies indicate that the use of mannitol in patients with >2 exacerbations showed significant improvement in the St George’s Respiratory Questionnaire (SGRQ) score; however, there is no evidence of a reduction in the number of exacerbations, and, in fact, mannitol increased the time of first exacerbation. We need further studies to determine the role of mucoactive therapy in bronchiectasis treatment and to test the use of oral mucoactive measures such as carbocisteine in bronchiectasis.

## 6. Conclusions

Bronchiectasis, traditionally seen as a purely neutrophil-driven disease, is now increasingly recognized as a syndrome encompassing multiple immune profiles, each associated with distinct clinical trajectories and therapeutic opportunities. Data on bronchiectasis pathogenesis and clinical outcomes created the need for an expansion of the traditional distinction between neutrophilic and eosinophilic inflammation, such as the new endotype categorization by Choi et al. [71].

Neutrophilic inflammation remains the dominant pattern, characterized by excessive protease activity, impaired bacterial clearance, and high exacerbation burden. However, eosinophilic, mixed, and paucigranulocytic subgroups, as well as the role of macrophage/lymphocyte-related inflammation, are increasingly described. Eosinophilic bronchiectasis is less common, with emerging evidence for ICS and biologic agent responsiveness. Mixed phenotypes, combining neutrophilic and Th2 pathways, appear particularly high-risk, with elevated exacerbation rates irrespective of prior history. Meanwhile, macrophage and lymphocyte involvement underscores the complexity of immune dysregulation, whereas the paucigranulocytic phenotype raises important questions about disease variability and immune regulation.

Recognition of these phenotypes has the potential to transform bronchiectasis care as more therapeutic options arise. Biomarkers such as NE, BEC, FeNO and NLR may guide patient stratification and treatment selection. Point-of-care assays (e.g., NEATstik) and blood-based markers are especially promising for clinical translation. Targeted therapies—such as brensocatib for neutrophil elastase inhibition, biologics for eosinophilic inflammation, and inhaled antibiotics for infection control—illustrate how precision medicine strategies could replace the current “one-size-fits-all” approach. Nevertheless, therapeutic decision-making remains challenging due to overlapping phenotypes and limited randomized controlled trial data.

Several challenges hinder the clinical application of phenotyping as inflammatory profiles might vary over time, specific biomarkers are still under investigation, and large clinical trials addressing the clinical importance of this classification are limited. To integrate phenotype into clinical practice, determination and prognosis of each phenotype is needed as well as standardization of biomarker thresholds. Deeper immunophenotyping beyond neutrophils and eosinophils might reveal novel therapeutic targets. The conclusions of this review should be taken into consideration through the prism of some limitations. This review is narrative and descriptive in nature, without the structured methodology of a systematic review. As such, it is limited by potential selection bias and the absence of quantitative synthesis. The available evidence on inflammatory phenotypes in bronchiectasis is derived largely from small, heterogeneous studies, and there remains a lack of large prospective or randomized controlled trials to validate proposed phenotypes and their therapeutic implications.

Advances in biomedical technologies hold considerable promises for refining bronchiectasis phenotyping and personalizing care. High-throughput sputum proteomics and metabolomics can provide detailed molecular signatures of airway inflammation, enabling stratification beyond traditional cell-based categories. AI-based imaging and machine learning algorithms may allow automated, reproducible assessment of radiological features, identifying subgroups with distinct clinical trajectories. Integration of these tools with clinical and microbiological data could transform the precision medicine framework in bronchiectasis, paving the way for individualized treatment strategies and targeted clinical trials.

Bronchiectasis is not a uniform disease but a heterogenous syndrome characterized by distinct inflammatory phenotypes. Understanding inflammatory phenotypes offers a path toward personalized treatment strategies and improved clinical outcomes in bronchiectasis. Moving from descriptive phenotypes towards integrated biomarker-driven phenotypes represents the next step toward true precision medicine in bronchiectasis.

## Figures and Tables

**Table 1:** Personalized treatments for inflammatory phenotypes of bronchiectasis.

Phenotype	Inflammatory Markers	Microbiological Profile	Associated Comorbidities	Clinical Features	Treatment
Neutrophilic	Sputum NE (NEATstik), sputum/BAL IL-8, NLR	*P. aeruginosa*, other Gram-negative bacteria	COPD, chronic infection	Most common; frequent exacerbations, worse lung function decline, severe progression, systemic inflammation	Inhaled antibiotics, macrolides, targeted therapy—brensocatib, airway clearance
Eosinophilic	Blood eosinophil count, sputum EPX, FeNO, IL-5/IL-13	Often culture-negative, *Aspergillus* (ABPA)	Asthma, ABPA, COPD	Heterogeneous prognosis; bronchodilator reversibility, ICS responsive subset	Inhaled Corticosteroids, biologic agents: anti-IL-5 (mepolizumab), anti-IL-5Rα (benralizumab)
Mixed	Combined cytokine profiles (IL-8, IL-5), FeNO, sputum proteases	Often *P. aeruginosa*, reduced microbial diversity	-	Dual inflammation	Combination of ICS, biologic agents and antibiotics based on patient’s characteristics
Paucigranulocytic	No specific biomarkers; low neutrophils/eosinophils	-	-	Milder clinical manifestations, transitional state (?)	Supportive therapy, airway clearance, treatment of underlying cause

## Data Availability

No new data was created. Data sharing is not applicable to this article.

## References

[1] Polverino E., Goeminne P.C., McDonnell M.J., Aliberti S., Marshall S.E., Loebinger M.R., Murris M., Cantón R., Torres A., Dimakou K. (2017). European Respiratory Society guidelines for the management of adult bronchiectasis. Eur. Respir. J..

[2] Flume P.A., Chalmers J.D., Olivier K.N. (2018). Advances in bronchiectasis: Endotyping, genetics, microbiome, and disease heterogeneity. Lancet.

[3] Chalmers J.D., Chang A.B., Chotirmall S.H., Dhar R., McShane P.J. (2018). Bronchiectasis. Nat. Rev. Dis. Prim..

[4] Chalmers J.D., Polverino E., Crichton M.L., Ringshausen F.C., De Soyza A., Vendrell M., Burgel P.R., Haworth C.S., Loebinger M.R., Dimakou K. (2023). Bronchiectasis in Europe: Data on disease characteristics from the European Bronchiectasis registry (EMBARC). Lancet Respir. Med..

[5] Barbosa M., Chalmers J.D. (2023). Bronchiectasis. Presse Méd..

[6] Mishra M., Kumar N., Jaiswal A., Verma A., Kant S. (2012). Kartagener′s syndrome: A case series. Lung India.

[7] Schäfer J., Griese M., Chandrasekaran R., Chotirmall S.H., Hartl D. (2018). Pathogenesis, imaging and clinical characteristics of CF and non-CF bronchiectasis. BMC Pulm. Med..

[8] Zhou W., Li Y., Zheng H., He M., Zhang M., Chen Q., Situ C., Wang Y., Zhang T., Chen K. (2025). Whole exome sequencing enhances diagnosis of hereditary bronchiectasis. Orphanet J. Rare Dis..

[9] Gao Y., Guan W., Liu S., Wang L., Cui J., Chen R., Zhang G. (2016). Aetiology of bronchiectasis in adults: A systematic literature review. Respirology.

[10] Gómez-Olivas J.D., Oscullo G., Martínez-García M.Á. (2023). Etiology of Bronchiectasis in the World: Data from the Published National and International Registries. J. Clin. Med..

[11] Tunney M.M., Einarsson G.G., Wei L., Drain M., Klem E.R., Cardwell C., Ennis M., Boucher R.C., Wolfgang M.C., Elborn J.S. (2013). Lung Microbiota and Bacterial Abundance in Patients with Bronchiectasis when Clinically Stable and During Exacerbation. Am. J. Respir. Crit. Care Med..

[12] Metersky M.L., Aksamit T.R., Barker A., Choate R., Daley C.L., Daniels L.A., DiMango A., Eden E., Griffith D., Johnson M. (2018). The Prevalence and Significance of *Staphylococcus aureus* in Patients with Non–Cystic Fibrosis Bronchiectasis. Ann. Am. Thorac. Soc..

[13] Sisodia J., Bajaj T. (2025). Allergic Bronchopulmonary Aspergillosis. StatPearls [Internet].

[14] Martínez García M.Á., Soriano J.B. (2022). Asthma, Bronchiectasis, and Chronic Obstructive Pulmonary disease: The Bermuda Triangle of the Airways. Chin. Med. J..

[15] King P. (2009). The pathophysiology of bronchiectasis. Int. J. Chronic Obstr. Pulm. Dis..

[16] Cole P.J. (1986). Inflammation: A two-edged sword-the model of bronchiectasis. Eur. J. Respir. Dis. Suppl..

[17] Keir H.R., Chalmers J.D. (2021). Pathophysiology of Bronchiectasis. Semin. Respir. Crit. Care Med..

[18] Raboso B., Pou C., Abril R., Erro M., Sánchez C., Manzano C., Zamarrón E., Suarez-Cuartin G., González J. (2024). Bronchiectasis. Open Respir. Arch..

[19] Bush A., Floto R.A. (2019). Pathophysiology, causes and genetics of paediatric and adult bronchiectasis. Respirology.

[20] Solarat B., Perea L., Faner R., De La Rosa D., Martínez-García M.Á., Sibila O. (2023). Pathophysiology of Chronic Bronchial Infection in Bronchiectasis. Arch. Bronconeumol..

[21] Chalmers J.D., Smith M.P., McHugh B.J., Doherty C., Govan J.R., Hill A.T. (2012). Short- and Long-Term Antibiotic Treatment Reduces Airway and Systemic Inflammation in Non–Cystic Fibrosis Bronchiectasis. Am. J. Respir. Crit. Care Med..

[22] Perea L., Faner R., Chalmers J.D., Sibila O. (2024). Pathophysiology and genomics of bronchiectasis. Eur. Respir. Rev..

[23] Keir H.R., Shoemark A., Dicker A.J., Perea L., Pollock J., Giam Y.H., Suarez-Cuartin G., Crichton M.L., Lonergan M., Oriano M. (2021). Neutrophil extracellular traps, disease severity, and antibiotic response in bronchiectasis: An international, observational, multicohort study. Lancet Respir. Med..

[24] Angrill J., Agustí C., De Celis R., Filella X., Rañó A., Elena M., De La Bellacasa J.P., Xaubet A., Torres A. (2001). Bronchial Inflammation and Colonization in Patients with Clinically Stable Bronchiectasis. Am. J. Respir. Crit. Care Med..

[25] Bedi P., Davidson D.J., McHugh B.J., Rossi A.G., Hill A.T. (2018). Blood Neutrophils Are Reprogrammed in Bronchiectasis. Am. J. Respir. Crit. Care Med..

[26] Reynolds C.J., Quigley K., Cheng X., Suresh A., Tahir S., Ahmed-Jushuf F., Nawab K., Choy K., Walker S.A., Mathie S.A. (2018). Lung Defense Through IL-8 Carries a Cost of Chronic Lung Remodeling and Impaired Function. Am. J. Respir. Cell Mol. Biol..

[27] Watt A.P., Brown V., Courtney J., Kelly M., Garske L., Elborn J.S., Ennis M. (2004). Neutrophil apoptosis, proinflammatory mediators and cell counts in bronchiectasis. Thorax.

[28] Martins M., Keir H.R., Chalmers J.D. (2023). Endotypes in bronchiectasis: Moving towards precision medicine. A narrative review. Pulmonology.

[29] Chan S.C.H., Shum D.K.Y., Ip M.S.M. (2003). Sputum Sol Neutrophil Elastase Activity in Bronchiectasis: Differential Modulation by Syndecan-1. Am. J. Respir. Crit. Care Med..

[30] Voynow J.A., Young L.R., Wang Y., Horger T., Rose M.C., Fischer B.M. (1999). Neutrophil elastase increases *MUC5AC* mRNA and protein expression in respiratory epithelial cells. Am. J. Physiol. Lung Cell. Mol. Physiol..

[31] Amati F., Simonetta E., Gramegna A., Tarsia P., Contarini M., Blasi F., Aliberti S. (2019). The biology of pulmonary exacerbations in bronchiectasis. Eur. Respir. Rev..

[32] Chalmers J.D., Moffitt K.L., Suarez-Cuartin G., Sibila O., Finch S., Furrie E., Dicker A., Wrobel K., Elborn J.S., Walker B. (2017). Neutrophil Elastase Activity Is Associated with Exacerbations and Lung Function Decline in Bronchiectasis. Am. J. Respir. Crit. Care Med..

[33] Dubois A.V., Gauthier A., Bréa D., Varaigne F., Diot P., Gauthier F., Attucci S. (2012). Influence of DNA on the Activities and Inhibition of Neutrophil Serine Proteases in Cystic Fibrosis Sputum. Am. J. Respir. Cell Mol. Biol..

[34] Shoemark A., Cant E., Carreto L., Smith A., Oriano M., Keir H.R., Perea L., Canto E., Terranova L., Vidal S. (2019). A point-of-care neutrophil elastase activity assay identifies bronchiectasis severity, airway infection and risk of exacerbation. Eur. Respir. J..

[35] Dente F.L., Bilotta M., Bartoli M.L., Bacci E., Cianchetti S., Latorre M., Malagrinò L., Nieri D., Roggi M.A., Vagaggini B. (2015). Neutrophilic Bronchial Inflammation Correlates with Clinical and Functional Findings in Patients with Noncystic Fibrosis Bronchiectasis. Mediat. Inflamm..

[36] Finch S., McDonnell M.J., Abo-Leyah H., Aliberti S., Chalmers J.D. (2015). A Comprehensive Analysis of the Impact of *Pseudomonas aeruginosa* Colonisation on Prognosis in Adult Bronchiectasis. Ann. Am. Thorac. Soc..

[37] Martinez-Garcia M.A., Athanazio R.A., Girón R.M., Máiz-Carro L., De La Rosa D., Olveira C., De Gracia J., Vendrell M., Prados-Sánchez C., Gramblicka G. (2017). Predicting high risk of exacerbations in bronchiectasis: The E-FACED score. Int. J. Chron. Obstruct. Pulmon. Dis..

[38] Vidaillac C., Chotirmall S.H. (2021). *Pseudomonas aeruginosa* in bronchiectasis: Infection, inflammation, and therapies. Expert Rev. Respir. Med..

[39] Araújo D., Shteinberg M., Aliberti S., Goeminne P.C., Hill A.T., Fardon T.C., Obradovic D., Stone G., Trautmann M., Davis A. (2018). The independent contribution of *Pseudomonas aeruginosa* infection to long-term clinical outcomes in bronchiectasis. Eur. Respir. J..

[40] Pieters A., Bakker M., Hoek R.A.S., Altenburg J., Van Westreenen M., Aerts J.G.J.V., Van Der Eerden M.M. (2019). Predicting factors for chronic colonization of *Pseudomonas aeruginosa* in bronchiectasis. Eur. J. Clin. Microbiol. Infect. Dis..

[41] Martínez-García M.A., Soler-Cataluña J.-J., Perpiñá-Tordera M., Román-Sánchez P., Soriano J. (2007). Factors Associated with Lung Function Decline in Adult Patients with Stable Non-Cystic Fibrosis Bronchiectasis. Chest.

[42] Chalmers J.D., Elborn S., Greene C.M. (2023). Basic, translational and clinical aspects of bronchiectasis in adults. Eur. Respir. Rev..

[43] Kavanaugh J.S., Flack C.E., Lister J., Ricker E.B., Ibberson C.B., Jenul C., Moormeier D.E., Delmain E.A., Bayles K.W., Horswill A.R. (2019). Identification of Extracellular DNA-Binding Proteins in the Biofilm Matrix. mBio.

[44] Chalmers J.D., Aliberti S., Filonenko A., Shteinberg M., Goeminne P.C., Hill A.T., Fardon T.C., Obradovic D., Gerlinger C., Sotgiu G. (2018). Characterization of the “Frequent Exacerbator Phenotype” in Bronchiectasis. Am. J. Respir. Crit. Care Med..

[45] Rademacher J., Welte T. (2011). Bronchiectasis. Dtsch. Ärztebl. Int..

[46] Gao Y., Richardson H., Dicker A.J., Barton A., Kuzmanova E., Shteinberg M., Perea L., Goeminne P.C., Cant E., Hennayake C. (2024). Endotypes of Exacerbation in Bronchiectasis: An Observational Cohort Study. Am. J. Respir. Crit. Care Med..

[47] Baker C., Chalmers J.D. (2025). Viruses in bronchiectasis. ERJ Open Res..

[48] Martinez-Garcia M.A., Sibila O., Aliberti S. (2022). Bronchiectasis: A pulmonary disease with systemic consequences. Respirology.

[49] Saleh A.D., Chalmers J.D., De Soyza A., Fardon T.C., Koustas S.O., Scott J., Simpson A.J., Brown J.S., Hurst J.R. (2017). The heterogeneity of systemic inflammation in bronchiectasis. Respir. Med..

[50] Zahorec R. (2021). Neutrophil-to-lymphocyte ratio, past, present and future perspectives. Bratisl. Lek. Listy.

[51] Georgakopoulou V.E., Trakas N., Damaskos C., Garmpis N., Karakou E., Chatzikyriakou R., Lambrou P., Tsiafaki X. (2020). Neutrophils to Lymphocyte Ratio as a Biomarker in Bronchiectasis Exacerbation: A Retrospective Study. Cureus.

[52] Tsikrika S., Dimakou K., Papaioannou A.I., Hillas G., Thanos L., Kostikas K., Loukides S., Papiris S., Koulouris N., Bakakos P. (2017). The role of non-invasive modalities for assessing inflammation in patients with non-cystic fibrosis bronchiectasis. Cytokine.

[53] Shoemark A., Shteinberg M., De Soyza A., Haworth C.S., Richardson H., Gao Y., Perea L., Dicker A.J., Goeminne P.C., Cant E. (2022). Characterization of Eosinophilic Bronchiectasis: A European Multicohort Study. Am. J. Respir. Crit. Care Med..

[54] Martínez-García M.Á., Méndez R., Olveira C., Girón R., García-Clemente M., Máiz L., Sibila O., Golpe R., Rodríguez-Hermosa J.L., Barreiro E. (2023). The U-Shaped Relationship Between Eosinophil Count and Bronchiectasis Severity. CHEST.

[55] Hammad H., Lambrecht B.N. (2021). The basic immunology of asthma. Cell.

[56] Brightling C., Greening N. (2019). Airway inflammation in COPD: Progress to precision medicine. Eur. Respir. J..

[57] Wechsler M.E., Munitz A., Ackerman S.J., Drake M.G., Jackson D.J., Wardlaw A.J., Dougan S.K., Berdnikovs S., Schleich F., Matucci A. (2021). Eosinophils in Health and Disease: A State-of-the-Art Review. Mayo Clin. Proc..

[58] Pollock J., Keir H.R., Shoemark A., Dicker A.J., Giam Y.H., Crichton M.L., Cant E., Huang J.T.J., Chalmers J.D. (2022). Proteomic Markers of Eosinophilic Inflammation and Disease Severity in Bronchiectasis. Am. J. Respir. Crit. Care Med..

[59] Rogers G.B., Zain N.M.M., Bruce K.D., Burr L.D., Chen A.C., Rivett D.W., McGuckin M.A., Serisier D.J. (2014). A Novel Microbiota Stratification System Predicts Future Exacerbations in Bronchiectasis. Ann. Am. Thorac. Soc..

[60] Dicker A.J., Lonergan M., Keir H.R., Smith A.H., Pollock J., Finch S., Cassidy A.J., Huang J.T.J., Chalmers J.D. (2021). The sputum microbiome and clinical outcomes in patients with bronchiectasis: A prospective observational study. Lancet Respir. Med..

[61] Wang X., Villa C., Dobarganes Y., Olveira C., Girón R., García-Clemente M., Máiz L., Sibila O., Golpe R., Menéndez R. (2021). Phenotypic Clustering in Non-Cystic Fibrosis Bronchiectasis Patients: The Role of Eosinophils in Disease Severity. Int. J. Environ. Res. Public Health.

[62] Dai Z., Zhong Y., Cui Y., Ma Y., Zeng H., Chen Y. (2024). Analysis of clinical characteristics, prognosis and influencing factors in patients with bronchiectasis-chronic obstructive pulmonary disease overlap syndrome: A prospective study for more than five years. J. Glob. Health.

[63] Huang J.T.-J., Cant E., Keir H.R., Barton A.K., Kuzmanova E., Shuttleworth M., Pollock J., Finch S., Polverino E., Bottier M. (2022). Endotyping Chronic Obstructive Pulmonary Disease, Bronchiectasis, and the “Chronic Obstructive Pulmonary Disease-Bronchiectasis Association”. Am. J. Respir. Crit. Care Med..

[64] Oscullo G., Gómez-Olivas J.D., Ingles M., Mompean S., Martinez-Perez R., Suarez-Cuartin G., la Rosa-Carrillo D., Martinez-Garcia M.A. (2023). Bronchiectasis-COPD Overlap Syndrome: Role of Peripheral Eosinophil Count and Inhaled Corticosteroid Treatment. J. Clin. Med..

[65] Crimi C., Ferri S., Campisi R., Crimi N. (2020). The Link between Asthma and Bronchiectasis: State of the Art. Respiration.

[66] Polverino E., Dimakou K., Traversi L., Bossios A., Haworth C.S., Loebinger M.R., De Soyza A., Vendrell M., Burgel P.-R., Mertsch P. (2024). Bronchiectasis and asthma: Data from the European Bronchiectasis Registry (EMBARC). J. Allergy Clin. Immunol..

[67] Ferri S., Crimi C., Campisi R., Cacopardo G., Paoletti G., Puggioni F., Crimi N., Heffler E. (2022). Impact of asthma on bronchiectasis severity and risk of exacerbations. J. Asthma.

[68] Zhang P.-A., Wang J.-L., Fu S.-Y., Luo H.-L., Qin R.-D., Li J. (2025). Mediators of the association between allergic diseases and bronchiectasis: A bi-directional univariable and multivariable Mendelian randomization study and mediation analysis. World Allergy Organ. J..

[69] Pollock J., Goeminne P.C., Aliberti S., Polverino E., Crichton M.L., Ringshausen F.C., Dhar R., Vendrell M., Burgel P.-R., Haworth C.S. (2025). Aspergillus Serologic Findings and Clinical Outcomes in Patients with Bronchiectasis. Chest.

[70] Sehgal I.S., Muthu V., Dhooria S., Prasad K.T., Garg M., Rudramurthy S.M., Aggarwal A.N., Chakrabarti A., Agarwal R. (2025). Impact of Bronchiectasis Severity on Clinical Outcomes in Patients with Allergic Bronchopulmonary Aspergillosis: A Retrospective Cohort Study. J. Allergy Clin. Immunol. Pract..

[71] Choi H., Ryu S., Keir H.R., Giam Y.H., Dicker A.J., Perea L., Richardson H., Huang J.T.J., Cant E., Blasi F. (2023). Inflammatory Molecular Endotypes in Bronchiectasis: A European Multicenter Cohort Study. Am. J. Respir. Crit. Care Med..

[72] Ntontsi P., Loukides S., Bakakos P., Kostikas K., Papatheodorou G., Papathanassiou E., Hillas G., Koulouris N., Papiris S., Papaioannou A.I. (2017). Clinical, functional and inflammatory characteristics in patients with paucigranulocytic stable asthma: Comparison with different sputum phenotypes. Allergy.

[73] Papaioannou A.I., Fouka E., Ntontsi P., Stratakos G., Papiris S. (2022). Paucigranulocytic Asthma: Potential Pathogenetic Mechanisms, Clinical Features and Therapeutic Management. J. Pers. Med..

[74] Shoemark A., Devaraj A., Meister M., Ozerovitch L., Hansell D.M., Wilson R. (2011). Elevated peripheral airway nitric oxide in bronchiectasis reflects disease severity. Respir. Med..

[75] Fuschillo S., De Felice A., Balzano G. (2008). Mucosal inflammation in idiopathic bronchiectasis: Cellular and molecular mechanisms. Eur. Respir. J..

[76] Zheng L., Shum H., Tipoe G.L., Leung R., Lam W.K., Ooi G.C., Tsang K.W. (2001). Macrophages, neutrophils and tumour necrosis factor-α expression in bronchiectatic airways in vivo. Respir. Med..

[77] Fouka E., Lamprianidou E., Arvanitidis K., Filidou E., Kolios G., Miltiades P., Paraskakis E., Antoniadis A., Kotsianidis I., Bouros D. (2014). Low-dose clarithromycin therapy modulates Th17 response in non-cystic fibrosis bronchiectasis patients. Lung.

[78] Frija-Masson J., Martin C., Regard L., Lothe M.-N., Touqui L., Durand A., Lucas B., Damotte D., Alifano M., Fajac I. (2017). Bacteria-driven peribronchial lymphoid neogenesis in bronchiectasis and cystic fibrosis. Eur. Respir. J..

[79] Gaga M., Bentley A.M., Humbert M., Barkans J., O’Brien F., Wathen C.G., Kay A.B., Durham S.R. (1998). Increases in CD4+ T lymphocytes, macrophages, neutrophils and interleukin 8 positive cells in the airways of patients with bronchiectasis. Thorax.

[80] Okabayashi H., Baba T., Ootoshi R., Shintani R., Tabata E., Ikeda S., Niwa T., Oda T., Okuda R., Sekine A. (2020). Evaluation of lymphocytic infiltration in the bronchial glands of Sjögren’s syndrome in transbronchial lung cryobiopsy. BMC Pulm. Med..

[81] Silva J.R., Jones J.A., Cole P.J., Poulter L.W. (1989). The immunological component of the cellular inflammatory infiltrate in bronchiectasis. Thorax.

[82] 82. Eller J., Silva J.R.L.E., Poulter L.W., Lode H., Cole P.J. (1994). Cells and Cytokines in Chronic Bronchial Infection: CELLS & CYTOKINES. Ann. N. Y. Acad. Sci..

[83] Fouka E., Lindén A., Bossios A. (2025). The role of T-helper and T regulatory cells in driving neutrophilic and eosinophilic inflammation in bronchiectasis. Front. Immunol..

[84] Altenburg J., de Graaff C.S., Stienstra Y., Sloos J.H., van Haren E.H.J., Koppers R.J.H., van der Werf T.S., Boersma W.G. (2013). Effect of azithromycin maintenance treatment on infectious exacerbations among patients with non-cystic fibrosis bronchiectasis: The BAT randomized controlled trial. JAMA.

[85] Wong C., Jayaram L., Karalus N., Eaton T., Tong C., Hockey H., Milne D., Fergusson W., Tuffery C., Sexton P. (2012). Azithromycin for prevention of exacerbations in non-cystic fibrosis bronchiectasis (EMBRACE): A randomised, double-blind, placebo-controlled trial. Lancet.

[86] Chalmers J.D., Aliberti S., Blasi F. (2015). Management of bronchiectasis in adults. Eur. Respir. J..

[87] Cordeiro R., Choi H., Haworth C.S., Chalmers J.D. (2024). The Efficacy and Safety of Inhaled Antibiotics for the Treatment of Bronchiectasis in Adults: Updated Systematic Review and Meta-Analysis. Chest.

[88] Haworth C.S., Bilton D., Chalmers J.D., Davis A.M., Froehlich J., Gonda I., Thompson B., Wanner A., O’Donnell A.E. (2019). Inhaled liposomal ciprofloxacin in patients with non-cystic fibrosis bronchiectasis and chronic lung infection with *Pseudomonas aeruginosa* (ORBIT-3 and ORBIT-4): Two phase 3, randomised controlled trials. Lancet Respir. Med..

[89] De Soyza A., Aksamit T., Bandel T.-J., Criollo M., Elborn J.S., Operschall E., Polverino E., Roth K., Winthrop K.L., Wilson R. (2018). RESPIRE 1: A phase III placebo-controlled randomised trial of ciprofloxacin dry powder for inhalation in non-cystic fibrosis bronchiectasis. Eur. Respir. J..

[90] Pollock J., Polverino E., Dhar R., Dimakou K., Traversi L., Bossios A., Haworth C., Loebinger M.R., De Soyza A., Vendrell M. (2025). Use of inhaled corticosteroids in bronchiectasis: Data from the European Bronchiectasis Registry (EMBARC). Thorax.

[91] Martínez-García M.Á., Oscullo G., García-Ortega A., Matera M.G., Rogliani P., Cazzola M. (2022). Inhaled Corticosteroids in Adults with Non-cystic Fibrosis Bronchiectasis: From Bench to Bedside. A Narrative Review. Drugs.

[92] Håkansson K.E.J., Fjaellegaard K., Browatzki A., Dönmez Sin M., Ulrik C.S. (2021). Inhaled Corticosteroid Therapy in Bronchiectasis is Associated with All-Cause Mortality: A Prospective Cohort Study. Int. J. Chronic Obstr. Pulm. Dis..

[93] Rademacher J., Konwert S., Fuge J., Dettmer S., Welte T., Ringshausen F.C. (2020). Anti-IL5 and anti-IL5Rα therapy for clinically significant bronchiectasis with eosinophilic endotype: A case series. Eur. Respir. J..

[94] Chalmers J.D., Burgel P.-R., Daley C.L., De Soyza A., Haworth C.S., Mauger D., Loebinger M.R., McShane P.J., Ringshausen F.C., Blasi F. (2025). Phase 3 Trial of the DPP-1 Inhibitor Brensocatib in Bronchiectasis. N. Engl. J. Med..

[95] Bell S.C., Grimwood K. (2025). Brensocatib in Bronchiectasis—A New Sheriff in Town?. N. Engl. J. Med..

[96] Chalmers J.D., Haworth C.S., Metersky M.L., Loebinger M.R., Blasi F., Sibila O., O’Donnell A.E., Sullivan E.J., Mange K.C., Fernandez C. (2020). Phase 2 Trial of the DPP-1 Inhibitor Brensocatib in Bronchiectasis. N. Engl. J. Med..

[97] Cipolla D., Zhang J., Korkmaz B., Chalmers J.D., Basso J., Lasala D., Fernandez C., Teper A., Mange K.C., Perkins W.R. (2023). Dipeptidyl peptidase-1 inhibition with brensocatib reduces the activity of all major neutrophil serine proteases in patients with bronchiectasis: Results from the WILLOW trial. Respir. Res..

[98] Chalmers J.D., Loebinger M.R., Teper A., McShane P.J., Fernandez C., Fucile S., Haworth C.S., Lauterio M., Van Der Laan R., Shih V.H. (2025). Brensocatib in patients with bronchiectasis: Subgroup analyses from the WILLOW trial. ERJ Open Res..

[99] Chalmers J.D., Metersky M.L., Feliciano J., Fernandez C., Teper A., Maes A., Hassan M., Chatterjee A. (2023). Benefit−risk assessment of brensocatib for treatment of non-cystic fibrosis bronchiectasis. ERJ Open Res..

[100] Ramsey K.A., Chen A.C.H., Radicioni G., Lourie R., Martin M., Broomfield A., Sheng Y.H., Hasnain S.Z., Radford-Smith G., Simms L.A. (2020). Airway Mucus Hyperconcentration in Non–Cystic Fibrosis Bronchiectasis. Am. J. Respir. Crit. Care Med..

